# Evaluation of the learning curve for transurethral plasmakinetic enucleation and resection of prostate using a mentor-based approach

**DOI:** 10.1590/S1677-5538.IBJU.2016.0237

**Published:** 2017

**Authors:** Lang Feng, Jian Song, Daoxin Zhang, Ye Tian

**Affiliations:** 1 Department of Urology, Beijing Friendship Hospital, Capital Medical University, Beijing, China

**Keywords:** Prostate, Transurethral Resection of Prostate

## Abstract

**Objective:**

To analyze the mentor-based learning curve of one single surgeon with transurethral plasmakinetic enucleation and resection of prostate (PKERP) prospectively.

**Materials and Methods:**

Ninety consecutive PKERP operations performed by one resident under the supervision of an experienced endourologist were studied. Operations were analyzed in cohorts of 10 cases to determine when a plateau was reached for the variables such as operation efficiency, enucleation efficiency and frequency of mentor advice (FMA). Patient demographic variables, perioperative data, complications and 12-month follow-up data were analyzed and compared with the results of a senior urologist.

**Results:**

The mean operative efficiency and enucleation efficiency increased from a mean of 0.49±0.09g/min and 1.11±0.28g/min for the first 10 procedures to a mean of 0.63±0.08g/min and 1.62±0.36g/min for case numbers 31-40 (p=0.003 and p=0.002). The mean value of FMA decreased from a mean of 6.7±1.5 for the first 10 procedures to a mean of 2.8±1.2 for case numbers 31-40 (p<0.01). The senior urologist had a mean operative efficiency and enucleation efficiency equivalent to those of the senior resident after 40 cases. There was significant improvement in 3, 6 and 12 month’s parameter compared with preoperative values (p<0.001).

**Conclusions:**

PKERP can be performed safely and efficiently even during the initial learning curve of the surgeon when closely mentored. Further well-designed trials with several surgeons are needed to confirm the results.

## INTRODUCTION

Transurethral resection of prostate (TURP) is the gold standard operation for symptomatic benign prostatic hyperplasia (BPH), but the complications such as transurethral resection syndrome (TURS) and blood loss still remain a problem, especially for the monopolar TURP with large prostates (1-3). Although there has been improvement in the TURP technique such as using the laser in the surgical treatment of BPH, the learning curve for the laser operation is longer and difficult to learn in a short period (4-6). Moreover, the expense of the laser may be higher and may not be widely available especially in the underdeveloped areas (7). Bipolar plasmakinetic TURP permits a longer operation time by saline irrigation instead of a mannitol solution, which significantly decreases the incidence of TURS (8). Plasmakinetic enucleation of prostate (PKEP) was developed to enucleate the prostate adenoma with the electrode loop and resectoscope tip without supernumerary equipment. It is considered an effective alternative to TURP and superior to monopolar TURP and bipolar TURP but with lower morbidity and shorter hospital stay (9, 10). Compared with holmium laser enucleation of the prostate (HoLEP), PKEP is also an effective and safe treatment for BPH (9). Consequently, PKEP has aroused a great deal of interest in the urological community, and efforts to learn and adopt this technique are being made. Since the separate commercial morcellator is not widely available in China, the enucleaton tissue can be resected with an electrode loop without other additional instruments, which was used in our study as transurethral plasmakinetic enucleation and resection of prostate (PKERP). To our best knowledge, there is only one published retrospective study that had evaluated the learning curve of PKERP (11) and no prospective trials have been published by now. Thus, the learning curve of this procedure has not been clearly defined. In this study, we evaluated the learning curve of a resident in PKERP and compared experience with a senior urologist.

## PATIENTS AND METHODS

### Study design

All operative procedures were performed in our institution by a single senior resident (surgeon A) who had received adequate endourological training. Surgeon A had finished one year of basic endourological training in our hospital and passed the examination. He had previously performed 80 transurethral resections of bladder tumours. However, he was inexperienced with transurethral prostate surgery, and had previously only performed about 10 TURP cases. An experienced urologist (surgeon B) was the expert in PKERP and had performed more than 300 PKERP procedures at our institution since its inception. He served as a mentor for surgeon A. Surgeon A had not previously performed PKERP, thus he familiarized himself with the PKERP technique by viewing videos of surgeon B performing the procedure. The PKERP technique and the videos were then reviewed with surgeon B to discuss remaining questions. Surgeon A assisted surgeon B in 10 PKERP procedures, and participated during enucleation and resection. These 10 patients were excluded from the analysis. When he was judged to be reasonably confident with the technical dynamics, a prospective study was designed to assess his progress in learning PKERP. All patients gave written informed consent and the study protocol was approved by the Institutional Review Board of our hospital in compliance with the Declaration of Helsinki.

A total of 90 consecutive patients with symptomatic BPH had undergone PKERP which were performed by surgeon A, with supervision by surgeon B. Surgeon B gave advice when it was necessary, but did not replace surgeon A unless safety issues emerged during the procedure. This parameter was recorded as frequency of mentor advice (FMA). Pitfalls, tips, and tricks of the PKERP procedure were discussed pre and post surgery in detail. The patient’s preoperative evaluations included transrectal ultrasonography with measurement of the total prostate size, serum prostate-specific antigen (PSA) assay and urine analysis. International Prostate Symptom Score (IPSS), quality of life (QoL) score, Qmax and postvoid residual (PVR) volume were recorded before and 3, 6, and 12 months after operation. Patients were excluded from the study if they had prostate cancer or neurogenic bladder or if they had undergone previous urethral or prostate surgery. Transperineal ultrasonography-guided prostate biopsies were performed to exclude prostate cancer when clinically necessary.

Data were collected during PKERP and included the total operative time, enucleation time, resection time, resected weight and FMA. The total operation time was defined as the interval between introducing the resectoscope and inserting the catheter. Operation, enucleation, and resection efficiency were calculated. The time to catheter removal and hospital stay were recorded after the operation. Complications were classified using the modified Clavien-Dindo classification of surgical complications and the data were also recorded.

To assess the number of procedures required to achieve competence in PKERP, the patients were first analyzed by divided with 10 cases depend on the time sequence. To assess the effect of the learning curve on the procedures outcome and complications, the 90 patients treated by surgeon A were divided into two subgroups according to the competence time (Group1: patients 1-40; Group2: patients 41-90). The results of surgeon A were also compared with those from a cohort of 40 consecutive PKERP procedures performed by the department’s senior urologist (surgeon C) during the study period, which formed a third group (Group 3). Surgeon C had more than 5 years of experience in PKERP surgery, and had performed more than 150 PKERP procedures.

### Statistical analysis

Statistical analysis was performed using the statistical software program SPSS (SPSS, Inc., Chicago, IL, version 16.0) for Windows. Continuous variables were presented as the mean value±standard deviation and differences between group data were analyzed by one-way independent analysis of variance (ANOVA) for continuous variables. Differences with P values <0.05 were considered significant.

### Surgical Techniques

PKERP was performed as previously described by Liu C (10) and Rao et al. (12). The procedure was performed with a 27Fr resectoscope with the loop of the bipolar PK system. The PK system uses 160W for cutting and 80W for coagulation. Physiologic saline was used as irrigation fluid. The ureteral orifices, bladder neck and verumontanum were identified preoperatively and incision was start close to the verumontanum from the 5 to the 7 o’clock positions. These grooves were deepened to the level of the surgical capsule. The tip of the resectoscope sheath was then inserted into the groove, which pushed the lobe along the surgical capsule line to create the cleavage plane between the detached lobe and the capsule. The bipolar plasmakinetic loop moved in exactly the same plane as the surgeon’s index finger does when performing open prostatectomy. Middle lobe, left lobe, and right lobe were dissected off the surgical capsule in a retrograde fashion from the apex toward the bladder using the bipolar plasmakinetic loop with arrest of bleeding. The enucleated lobes were devascularized simultaneously but still attached at the bladder neck by a narrow pedicle (the “mushroom” technique (13)). The enucleation adenoma was resected into smaller prostatic chips by the plasmakinetic loop and extracted by Ellic. A 22Fr triple-lumen catheter was inserted and connected to straight drainage after the operation. Continuous bladder irrigation was necessary with physiologic saline and stopped when the urine cleared of hematuria. After catheter drainage became clear, bladder irrigation was stopped. If catheter drainage was still clear, the catheters were removed within 24 hours and the patients were then discharged from the hospital within 24h after decatheterisation.

## RESULTS

### Patients’ Demographic and Perioperative Characteristics

All patients were successfully treated with PKERP. There were no perioperative deaths and no subject was converted to open prostatectomy. Of note, surgeon B did not take over any PKERP procedure in the 90 consecutive surgeries performed by surgeon A. Baseline and perioperative data are reported in Table-1. There were no statistically significant differences among the three groups (Group1, Group2 and Group3) with respect to age, prostate size, PSA level, IPSS, QOL score, Qmax, PVR, catheter time and hospital stay (p>0.05). The resection time was lower and the resected weight was higher in group 3 compared with group1 and group2, but the differences had no statistic significance (p>0.05). There were significant differences among the three groups with respect to the operation time, enucleation time, operation efficiency, enucleation efficiency and resection efficiency (p<0.05). The value of FMA in group 2 was significantly lower than group1 (p<0.05).

**Table 1:** Patients’ demographic and perioperative characteristics.

Parameters	Group 1 (n=40)	Group 2 (n=50)	Group 3 (n=40)	P value
Age(years)	72.8±7.3	70.9±8.1	72.1±8.7	0.528
Prostate size (mL)	75.2±28.1	81.8±23.9	78.9±24.9	0.476
PSA(ng/dL)	4.19±1.78	4.68±2.24	4.39±1.92	0.512
IPSS	21.8±2.3	22.5±1.9	21.7±1.4	0.110
QoL(score)	3.8±0.7	3.7±0.6	3.9±0.6	0.670
Qmax(mL/s)	7.6±2.5	7.7±2.6	6.9±2.2	0.252
PVR(mL)	86.6±37.7	101.7±47.8	94.7±40.7	0.211
Operation time(min)	79.2±24.6	71.3±23.9	67.9±18.8	0.040*
Enucleation time (min)	33.2±10.4	29.0±9.4	28.3±9.5	0.022*
Resection time (min)	45.9±17.1	42.0±15.4	39.8±11.8	0.115
Resected weight (g)	48.3±17.5	44.1±13.9	48.2±20.4	0.323
Operation efficiency (g/min)	0.59±0.09	0.61±0.08	0.68±0.13	0.036*
Enucleation efficiency (g/min)	1.49±0.39	1.56±0.30	1.71±0.44	0.019*
Resection efficiency (g/min)	1.04±0.17	1.12±0.15	1.29±0.35	0.027*
FMA	4.1±3.0	2.1±2.2	NA	<0.01*
Catheter time (d)	2.3±1.2	2.3±1.3	2.0±1.1	0.378
Hospital stay (d)	2.9±1.2	3.0±1.4	2.8±1.1	0.728

Data presented as mean±standard deviation. *p<0.05

**PSA =** prostate-specific antigen; **IPSS =** International Prostate Symptom Score; **QoL =** quality of life; **PVR =** postvoid residual volume; **FMA =** frequency of mentor advice; **Group 1 =** surgeon A, cases 1-40; **Group 2 =** surgeon A, cases 41-90; **Group 3 =** surgeon C, cases 1-40; **NA=** not applicable.

### Follow-up Data

There were 5 (5/90, 5.6%) patients of surgeon A who were lost to follow-up. Eighty-five (85/90, 94.4%) completed the twelve months-follow-up. No patients in group 3 were excluded from the study. Table-2 lists changes in IPSS score, QOL, Qmax and PVR in the 3, 6 and 12 months after the operation. There were significant improvements in 3, 6 and 12 month’s parameters compared with preoperative values (p<0.001). There were no statistically significant differences among the three groups with respect to preoperative and postoperative values (p>0.05).

**Table 2:** Preoperative characteristics and follow-up data.

Parameters	Group 1 (n=40)	Group 2 (n=50)	Group 3 (n=40)	P-value
**IPSS**				
Preop	21.8±2.3	22.5±1.9	21.7±1.4	0.110
3 month	8.1±2.8	8.2±2.7	7.5±2.5	0.432
6 month	7.3±2.4	7.7±2.1	7.1±2.1	0.395
12 month	6.8±1.9	6.9±1.8	6.7±1.8	0.925
P value	<0.001*	<0.001*	<0.001*	
**QoL(score)**				
Preop	3.8±0.7	3.7±0.6	3.9±0.6	0.670
3 month	1.7±0.7	1.8±0.7	1.7±0.6	0.878
6 month	1.5±0.5	1.6±0.7	1.5±0.5	0.789
12 month	1.4±0.5	1.4±0.4	1.4±0.5	0.786
P value	<0.001*	<0.001*	<0.001*	
**Qmax(mL/s)**				
Preop	7.6±2.5	7.7±2.6	6.9±2.2	0.252
3 month	20.2±2.8	20.7±2.9	20.9±2.6	0.523
6 month	21.2±2.3	21.3±2.3	21.4±2.1	0.883
12 month	21.5±1.8	21.6±1.7	21.7±1.9	0.935
P value	<0.001*	<0.001*	<0.001*	
**PVR(mL)**				
Preop	86.6±37.7	101.7±47.8	94.7±40.7	0.211
3 month	11.1±4.2	10.9±4.5	10.3±3.7	0.647
6 month	10.0±2.8	9.9±3.6	9.3±3.1	0.549
12 month	9.4±2.3	9.6±2.7	9.2±3.1	0.796
P value	<0.001*	<0.001*	<0.001*	

Data presented as mean±standard deviation. *p<0.001

**Preop =** preoperative; **IPSS =** International Prostate Symptom Score; **QoL =** quality of life; **PVR =** postvoid residual; **Group 1 =** surgeon A, cases 1-40; **Group 2 =** surgeon A, cases 41-90; **Group 3 =** surgeon C, cases 1-40.

### Complications

Complications are listed in Table-3. Capsule perforation occurred in 3 (3/40, 7.5%) patients in group 1. Bladder mucosa damage occurred in 2 (2/40, 5%) patients in group 1. All the damages were mild and treated with catheterization for 3-5 days. Transient urinary incontinence occurred in 3 (3/40, 7.5%) in group 1 and 2 (2/50, 4%) in group 2 and 1 (1/40, 2.5%) in group 3. No patient developed stress urinary incontinence persistent for more than three months. Totally there were 4 (4/130, 3.1%) patients who required blood transfusion because anemia existed preoperatively. Urinary tract infection occurred in 4 (4/130, 3.1%) patients, which were treated with antibiotics. There were 2 (2/130, 1.5%) patients who needed re-catheterization due to acute urinary retention after catheter removal, but these patients could self-void after bladder training for 5-7 days. Hematuria needing reoperation was observed in 1(1/40, 2.5%) patient in group 1, which underwent transurethral electric coagulation. The other postoperative complications included urethral stricture occurring in one patient in group 1 and in group 2, both requiring urethrotomy. Bladder-neck contracture occurred in 1 (1/40, 2.5%) patient in the group 1, which required transurethral resection of bladder neck.

**Table 3:** Complications of PKERP according to the CLAVIEN-DINDO grade system.

Complications (n%)	Group 1 (n=40)	Group 2 (n=50)	Group 3 (n=40)
**Grade I**			
Capsule perforation	3(7.5%)	0	0
Bladder mucosa damage	2(5%)	0	0
Transient incontinence	3(7.5%)	2(4%)	1(2.5%)
**Grade II**			
Blood transfusion	2(5%)	1(2%)	1(2.5%)
Urinary tract infection	2(5%)	1(2%)	1(2.5%)
Re-catheterization	1(2.5%)	1(2%)	0
**Grade IIIa**			
Hematuresis need reoperation	1(2.5%)	0	0
**Grade IIIb**			
Urethral stricture	1(2.5%)	1(2%)	0
Bladder-neck contracture	1(2.5%)	0	0

Data presented as n%.

**PKERP =** transurethral plasmakinetic enucleation and resection of prostate; **Group 1 =** surgeon A, cases 1-40; **Group 2 =** surgeon A, cases 41-90; **Group 3 =** surgeon C, cases 1-40.

### Learning Curve

**Figura 1:**
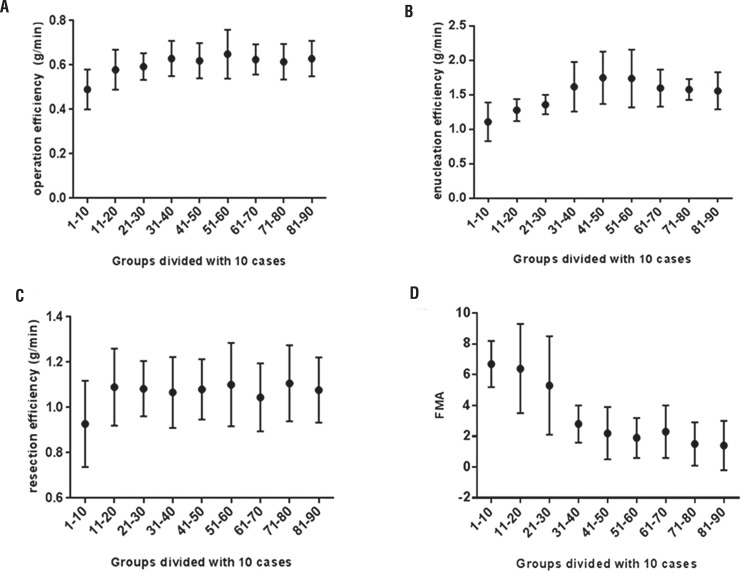
(A): Comparison of operation efficiency over the number of PKERP cases performed by surgeon A (n=90); (B): Comparison of enucleation efficiency over the number of PKERP cases performed by surgeon A (n=90); (C): Comparison of resection efficiency over the number of PKERP cases performed by surgeon A (n=90); (D): Comparison of FMA over the number of PKERP cases performed by surgeon A (n=90).

**Figura 2:**
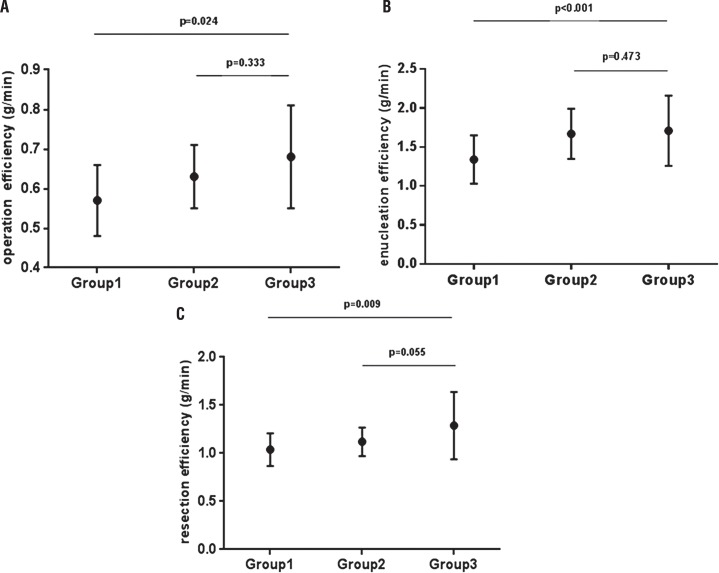
(A): Comparison of operation efficiency between the three groups; (B): Comparison of enucleation efficiency between the three groups; (C): Comparison of resection efficiency between the three groups.

In the 90 procedures performed by surgeon A, the mean operative efficiency and enucleation efficiency gradually increased (Figures-1A, 1B). They ranged from a mean of 0.49±0.09g/min and 1.11±0.28g/min for the first 10 PKERP procedures to a mean of 0.63±0.08g/min and 1.62±0.36g/min for case numbers 31-40. The increase in the mean operative efficiency and enucleation efficiency were statistically significant for the first 40 cases (p=0.003 and p=0.002). Then, few fluctuations were observed with respect to the mean operative efficiency and enucleation efficiency in the subsequent patient groups, indicating that a plateau had been reached (p=0.919 and p=0.232). Surgeon C had a mean operative efficiency of 0.68±0.13g/min and enucleation efficiency 1.71±0.44, which were similar to that achieved by surgeon A after 40 operations (Figure-2A and 2B; p=0.333 and p=0.473). The resection efficiency in surgeon A cases ranged from a mean of 0.91±0.09g/min for the first 10 PKERP procedures to a mean of 1.09±0.17g/min for case numbers 11-20. The increase in the mean resection efficiency was statistically significant for the first 20 cases (p=0.043). Then, few fluctuations were observed in the subsequent patient groups (Figure-1C, p=0.980). The resection efficiency in surgeon C was higher than surgeon A, however, the value had no statistic difference compared with group2 (Figure-2C, p=0.055). The value of FMA also reduced as experience in the procedure gradually increased (Figure-1D). The mean value of FMA was 6.7±1.5 for the first 10 cases, which decreased significantly to a mean value of 2.8±1.2 when the fortieth procedure had been completed (p<0.01). The value of FMA was maintained for case numbers 51-90 (group 2, p=0.246).

## DISCUSSION

HoLEP has been proven to be an effective, minimally invasive procedure for the surgical treatment of BPH (14). PKEP is as safe and effective as HoLEP according to the previously reports (9, 15), although the PKEP remains less versatile than the holmium laser, particularly in terms of stone disease; however, the lower capital costs and ease of use for this technique makes it a good choice for BPH (6, 11). To our best knowledge, there is no prospective trial which has evaluated the learning curve of PKERP procedure. In the present study, using a mentor-based approach, we present the first prospective analysis of individual learning curves of the PKERP procedure.

Ideally the learning curve for surgery is completed during residency or fellowship training. However, the opportunity is limited. The concern is that a patient will probably be unwilling to be treated by a novice surgeon due to concern about adverse outcomes. Thus, mentoring with an expert is a desirable method to be used to acquire the technique. For learning the technique of PKERP, the mentor-based approach can help the novice surgeon determine the correct tissue plane, comment on the depth of incision and encourages the resident to proceed if the appropriate maneuvers are being done (16). The video based education before mentor based training operation is also important. The novice surgeon can familiarized himself with the PKERP technique by viewing videos and reviewed the videos with mentor to discuss remaining questions. The detailed discussion of critical or problematic operative steps with an expert allows the novice to learn the pitfalls, and tips and tricks of the procedure, thus improving the quality of the PKERP operation. In the learning curve of HoLEP procedures with a mentor-based approach, El-Hakim and Elhilali (17) found that the outcome in 27 HoLEP procedures performed by a senior resident was comparable to that of 118 done by an experienced urologist. They concluded that extensive experience with transurethral surgery and the supervision by an experienced urologist were prerequisites for success. Our results also found that under the mentor supervision of an expert, the operation efficiency and enucleation efficiency of the resident were similar to that of a senior urologist after 40 cases. The resection weight of the prostate between the resident and the senior urologist were also similar. Using a mentor-based approach, a novice surgeon can perform PKERP with efficiency.

Compared with the monopolar TURP, the major benefits of bipolar TURP include the decreased elimination of dilutional hyponatremia even during longer operation time. The long safe operation time allows the beginner to recognize the anatomical landmarks meticulously and to enucleate the adenoma circumspectly. The anatomical landmarks that prompt the surgeon to identify the surgical plane mainly include capsule transverse fibers or fiber strands, capsule vessel reticula, capsule prostate calculi, and granular prostatic fluid retentates (10, 11). In retrograde fashion, the proximal and lateral margins of the verumontanum are the best sites for starting the enucleation of adenoma, where the plane between the surgical capsule and the hyperplasia adenoma is permanent (11). In our first 40 patients, more operation time and enucleation time were spent on identifying the anatomical landmarks. Therefore, the mean operation efficiency and enucleation efficiency was lower than in the later 50 cases.

In our series, there was a learning curve with this technique of at least 40 cases after the resident had experienced the procedure. The operation enucleation efficiency was increased to stationary after 40 cases. Our results were similar to the findings from another study. Xiong W. et al. (11) stated that an inexperienced endourologist in plasmakinetic prostate enucleation can reach an efficiency plateau after 50 cases. However, there was no tutoring or mentor supervision in their study. The present study also showed that the mentor advice decreased to a stationary low frequency after 40 cases. The resident could independently complete the operation without a proper instructor after 40 cases, which was in accordance with the increase of surgical experience. The resection efficiency reached an efficiency plateau after 20 cases, which was faster than the enucleation efficiency. The reason might be the resident had previous experience in endourology. Although there was no statistic difference between the later 50 cases and the senior urologist, the value of the resident was still lower than the senior urologist. The results indicated that with the increased experience, the resection efficiency could be further increased and the operation time could be shortened.

Three cases of capsule perforation occurred in the initial 40 cases due to the unfamiliar experience of the identification of the surgical plane between prostate adenoma and prostate capsule. The three cases of perforation were minor and did not alter the clinical course. There were some reports indicating that in smaller fibrotic prostates, the surgical capsule was often less distinct and the plane of dissection more difficult than in larger glands, in which the greater degree of peripheral compression tended to create a more easily identifiable plane (18, 19). We also would not recommend a patient with a small size prostate as the primary choice for a novice’s initial training. There were three bladder mucosa damage cases which occurred in the first 40 cases in this study. The enucleation tissue sometimes can affect the vision of the operator especially when the prostate volume is large and hematuria exists. The achievement of thorough hemostasis and bladder distension are essential to avoid this complication. In addition, no serious complications were experienced in our patients, and all transient urinary incontinence cases have completely recovered. At a 12 month’s follow-up, our results showed a quick and durable improvement in IPSS, QOL, Qmax and PVR after operation, which agreed with those previously published reports (20, 21). The clinical efficacy of PKERP performed by the resident was also comparable with those of the senior urologist. The results indicated that PKERP was a safe and efficient treatment for urologists, even for an inexperienced surgeon.

There are still some limitations in our study that should be considered. First, this study describes the PKERP learning curve for only one surgeon. He had experience in endourology and his learning curve may not be applicable to someone emerging from their residency or someone who has limited endourological training. However, to the best of our knowledge, we provided the first prospective analysis of individual learning curves of the PKERP procedure and showed its safety and effectiveness during the initial learning experience of the surgeon when closely mentored. The mentor-based approach is recommended for an inexperienced surgeon to study the PKERP procedure. Secondly, the average follow-up was too short to demonstrate the long-term efficacy. Larger sample trials including more surgeons with longer follow-up are needed to further confirm our results.

## CONCLUSIONS

The PKERP is a promising surgical treatment for safe and effective removal of prostatic tissue in patients with symptomatic BPH. PKERP can be performed safely and efficiently even during the initial learning curve of the surgeon when closely mentored. We found that performing the procedure in 40 cases is sufficient for a single operator to complete the learning curve. However, further well-designed trials with several surgeons are needed to confirm the results.

## ABBREVIATIONS

TURP: transurethral resection of prostate
TURS: transurethral resection syndrome
PKEP: plasmakinetic enucleation of prostate
HoLEP: holmium laser enucleation of the prostate
PKERP: transurethral plasmakinetic enucleation and resection of prostate
TURBT: transurethral resection of the bladder tumor
FMA: frequency of mentor advice
PSA: prostate specific antigen
IPSS: International Prostate Symptom Score
QoL: quality of life
PVR: postvoid residual
